# Left Atrial Appendage Thrombus Formation Despite Continuous Non-Vitamin K Antagonist Oral Anticoagulant Therapy in Atrial Fibrillation Patients Undergoing Electrical Cardioversion or Catheter Ablation: A Comparison of Dabigatran and Rivaroxaban

**DOI:** 10.1155/2020/1206402

**Published:** 2020-09-17

**Authors:** Iwona Gorczyca, Magdalena Chrapek, Olga Jelonek, Anna Michalska, Agnieszka Kapłon-Cieślicka, Beata Uziębło-Życzkowska, Monika Budnik, Monika Gawałko, Paweł Krzesiński, Agnieszka Jurek, Piotr Scisło, Janusz Kochanowski, Marek Kiliszek, Grzegorz Gielerak, Krzysztof J. Filipiak, Grzegorz Opolski, Beata Wożakowska-Kapłon

**Affiliations:** ^1^Collegium Medicum, The Jan Kochanowski University, Kielce 25-369, Poland; ^2^1^st^ Clinic of Cardiology and Electrotherapy, Swietokrzyskie Cardiology Centre, Kielce 25-736, Poland; ^3^Faculty of Natural Sciences, The Jan Kochanowski University, Kielce 25-369, Poland; ^4^1st Chair and Department of Cardiology, Medical University of Warsaw, Warsaw 02-097, Poland; ^5^Department of Cardiology and Internal Diseases, Military Institute of Medicine, Warsaw 04-141, Poland

## Abstract

Left atrial appendage thrombus (LAAT) may be detected by transesophageal echocardiography (TOE) in patients with atrial fibrillation (AF) despite continuous anticoagulation therapy. We examined the factors predisposing to LAAT in patients treated with the anticoagulants dabigatran and rivaroxaban. We retrospectively evaluated 1,256 AF patients from three centres who underwent TOE before electrical cardioversion (*n* = 611, 51.4%) or catheter ablation (*n* = 645, 48.6%) from January 2013 to December 2019 and had been on at least three weeks of continuous dabigatran (*n* = 603, 48%) or rivaroxaban (*n* = 653, 52%) therapy. Preprocedural TOE diagnosed LAAT in 51 patients (4.1%), including 30 patients (5%) treated with dabigatran and 21 patients (3.2%) treated with rivaroxaban (*p*=0.1145). In multivariate logistic regression, predictors of LAAT in patients treated with dabigatran were non-paroxysmal AF (vs. paroxysmal AF) (OR = 6.2, *p*=0.015), heart failure (OR = 3.22, *p*=0.003), and a eGFR <60 ml/min/1.73 m^2^ (OR = 2.65, *p*=0.012); the predictors in patients treated with rivaroxaban were non-paroxysmal AF (vs. paroxysmal AF) (OR = 5.73, *p*=0.0221) and heart failure (OR = 3.19, *p*=0.116). In ROC analysis of the dabigatran group, the area under the curve (AUC) for the CHA_2_DS_2_-VASc-RAF score was significantly higher (0.78) than those for the CHADS_2_, CHA_2_DS_2_-VASc, and R_2_CHADS_2_ scores (0.67, 0.70, and 0.72, respectively). In the rivaroxaban group, the CHA_2_DS_2_-VASc-RAF score also performed significantly better (AUC of 0.77) than the CHADS_2_, CHA_2_DS_2_-VASc, and R_2_CHADS_2_ scores (AUC of 0.66, 0.64, and 0.67, respectively). The risk of LAAT was the same for patients in both treatment groups. In all patients, non-paroxysmal AF or heart failure, and in patients treated with dabigatran an eGFR <60 ml/min/1.73 m^2^, were independent predictors of LAAT. The new CHA_2_DS_2_-VASc-RAF scale had the highest predictive value for LAAT in the entire study population.

## 1. Introduction

Risk factors for thromboembolic complications in patients with atrial fibrillation (AF) are well established [1]. Stroke, however, may result from different mechanisms, such as left atrial appendage thrombus (LAAT) embolisation—the risk factors for which are poorly understood and are not necessarily the same as those for stroke in AF patients [2].

According to applicable guidelines, preoperative oral anticoagulant (OAC) therapy is recommended for a minimum of three weeks to prevent periprocedural thromboembolism in AF patients scheduled for elective electrical cardioversion or catheter ablation; alternatively, transoesophageal echocardiography (TOE) may be used to exclude LAAT before the procedure [3]. OAC therapy reduced the periprocedural stroke and systemic embolism risk from 3.4% to <1% [4, 5]. Some patients had LAAT despite optimal anticoagulation therapy, but this did not necessarily explain the development of thromboembolism in AF patients. Many studies show that the frequency of LAAT in patients with AF varies depending on type and duration of anticoagulation therapy, type of AF, echocardiographic parameters, and concomitant diseases [6–9].

The non-vitamin K antagonist oral anticoagulants (NOAC) group includes the direct thrombin inhibitor dabigatran and the direct factor Xa (FXa) inhibitors apixaban, edoxaban, and rivaroxaban. These were approved for AF patients before electrical cardioversion and catheter ablation. The NOACs vary with respect to their mechanism of action, pharmacokinetics, and dosing; so, it is possible that their efficacies in LAAT prevention will differ [10].

Routine periprocedural assessment of NOAC compliance may be problematic because regular laboratory monitoring is not required; therefore, the routine use of TOE prior to cardioversion is discussed extensively.

We examined the effect of specific NOACs on the rates of LAAT and assessed the LAAT predictors in AF patients who had been on continuous anticoagulation therapy prior to electrical cardioversion or catheter ablation.

## 2. Materials and Methods

### 2.1. Study Design and Participants

We retrospectively evaluated 1,312 AF patients from three centres who underwent TOE before electrical cardioversion or catheter ablation from January 2013 to December 2019 and had been on at least three weeks of continuous NOAC therapy. Patients on apixaban (*n* = 56) were excluded from the study due to low numbers in this category. The final study population consisted of 1,256 patients.

Exclusion criteria were the presence of moderate or severe mitral valve stenosis or a mechanical heart valve.

Research protocol and retrospective review of medical records were approved by the ethics committees of each institution. As this was an observational, retrospective study, the ethics committees waived the requirement of obtaining informed consent from the patients.

All clinical, laboratory, and echocardiographic (including TOE) data were obtained retrospectively from medical records. Patients were divided into three groups according to the AF type (paroxysmal, persistent, and permanent AF), based on a thorough analysis of all available medical records. Patients were classified as having permanent AF after unsuccessful cardioversion during index hospitalisation, or if after receiving a previous diagnosis of permanent AF, their diagnosis was changed to long-standing persistent AF prior to electrical cardioversion or catheter ablation; these patients were classified as permanent AF to distinguish them from persistent AF patients with a presumably lower AF burden.

Vascular disease was defined as prior myocardial infarction, aortic plaque, or peripheral arterial disease.

The estimated glomerular filtration rate (eGFR) used to assess patients' kidney function was calculated using the Modification of Diet in Renal Disease Studyequation.

### 2.2. Evaluation of Thromboembolic Risk

The CHADS_2_, CHA_2_DS_2_-VASc, R_2_CHADS_2_, and CHA_2_DS_2_-VASc-RAF scales were used to estimate the risk of thromboembolic events in AF patients [11–14]. The CHA_2_DS_2_-VASc-RAF scale adds AF type and kidney function to the risk assessment according to the CHA_2_DS_2_-VASc scale. All scales used to estimate the risk of thromboembolic complications in AF patients are listed in Table 1. 34.5% of patients in the presented study was included in the Kapłon-Cieślicka [14] study upon which the CHA_2_DS_2_-VASc-RAF scale is based.

### 2.3. Management of Anticoagulation Therapy

All patients received continuous NOAC therapy (dabigatran or rivaroxaban) for at least three weeks before the TOE, including the day of the TOE. NOACs were dosed according to the manufacturer's recommendations.

### 2.4. Echocardiographic Evaluation

All TOE examinations were conducted within the 48 hours preceding the scheduled electrical cardioversion or catheter ablation procedures and were performed by certified echocardiographers (second-degree accreditation in echocardiography of the Section of Echocardiography of the Polish Cardiac Society (PCS)), using the General Electric Vivid 7 or E95 ultrasound system (General Electric, Milwaukee, WI), the EPIQ 7 ultrasound machine (Philips Medical Systems, Andover, MA), or the iE33 ultrasound machine (Philips Medical Systems) with the X72 t TOE ultrasound transducer (Philips Medical Systems).

LAAT was defined as an independently mobile echodense structure, distinct from the surrounding endocardium or pectinate muscles, and detected in more than one imaging plane. Dense spontaneous echocontrast (SEC) was defined as a dynamic “smoke-like” signal with a characteristic swirling motion or a dynamic gelatinous, precipitous echodensity without a discrete mass, present throughout the cardiac cycle.

When LAAT was suspected, the images were evaluated by two echocardiographers, and in some cases by a third echocardiographer, to establish an unanimous diagnosis and enable safe referral for electrical cardioversion or catheter ablation. Written informed consent for TOE was obtained from all patients.

In cases with confirmed LAAT, a decision against reversal of sinus rhythm was stated. Further procedures for these patients were decided upon individually.

### 2.5. Study Endpoint

The study endpoint was the presence of LAAT on TOE.

### 2.6. Statistical Analysis

Continuous data were described by means, standard deviations, medians, and interquartile range (IQR). Categorical data were summarized by frequencies and percentages. Group comparisons were performed using the chi-squared or Fisher exact test for categorical variables, the *t*-test for continuous, normally distributed variables, or the Mann–Whitney test for continuous, nonnormally variables (normality of distribution was checked with the Shapiro–Wilk test).

The LAAT occurrence was modelled by univariable and multivariable logistic regression in which the odds ratios (OR) and 95% confidence intervals (95% CI) were calculated.

The ability of predicting LAAT occurrence by CHADS_2_, CHA_2_DS_2_-VASc, CHA_2_DS_2_-VASc-RAF, and R_2_CHADS_2_ scoring systems was assessed by creating receiver operating characteristic (ROC) curves and area under the ROC curve (AUC). The optimal cutoff values were determined by maximising the Youden index. Sensitivity, specificity, and accuracy related to these cutoff values were also calculated, and comparison of ROC curves was performed by DeLong's test.

A two-tailed *p* value <0.05 was considered statistically significant. All statistical analyses were performed using the *R* software package version 3.6.2.

## 3. Results and Discussion

### 3.1. Characteristics of the Study Group

This study included 1,256 patients (61.9% male, mean age 62 years) referred to our centres for catheter ablation (*n* = 645, 48.6%) or electrical cardioversion (*n* = 611, 51.4%) of AF. All patients were on NOAC therapy: 603 patients (48%) on dabigatran and 653 patients (52%) on rivaroxaban. A reduced NOAC dose was taken by 110 patients (8.8%), 52 patients (8.6%) treated with dabigatran and 58 patients (8.9%) treated with rivaroxaban (*p*=0.8714). A comparison of baseline characteristics between patients treated with dabigatran and rivaroxaban is presented in Table 2.

According to the CHA_2_DS_2_-VASc scale, 841 patients (67%) had a high risk of thromboembolic events: 418 patients treated with dabigatran (69.3%) and 423 patients (64.8%) treated with rivaroxaban (*p*=0.1947). According to the HASBLED scale, 152 patients (12.1%) had a high risk of haemorrhagic complications: 83 patients (13.8%) treated with dabigatran and 69 patients (10.6%) treated with rivaroxaban (*p*=0.0826).

### 3.2. Prevalence of LAAT and SEC Detection by TOE

LAAT formation was found in 51 patients (4.1%), and there was no significant difference in incidence between patients treated with dabigatran vs. rivaroxaban (5% vs. 3.2%, *p*=0.1145) (Table 2). The incidence of SEC was lower in patients treated with dabigatran vs. rivaroxaban (12.6% vs. 18.8%, *p*=0.0025).

### 3.3. Analysis of Factors Predisposing to Thrombosis

In patients treated with dabigatran, those with LAAT were older than patients without LAAT, were more likely to be diabetic, had a higher HASBLED score, and had a lower GFR. Patients with LAAT—irrespective of which NOAC treatment they were on—were more likely to have heart failure, non-paroxysmal AF, and higher CHADS_2_, CHA_2_DS_2_-VASc, CHA_2_DS_2_-VASc-RAF, and R_2_CHADS scores than patients without LAAT (Table 2). Reduced doses of dabigatran and rivaroxaban yielded similar results in patients with and without LAAT (Table 3).

In multivariate logistic regression, predictors of LAAT in patients treated with dabigatran were heart failure, non-paroxysmal AF (vs paroxysmal AF), and an eGFR <60 ml/min/1.73 m^2^, as given in Table 4; predictors of LAAT in patients treated with rivaroxaban were heart failure and non-paroxysmal AF (vs paroxysmal AF), as given in Table 5.

### 3.4. Assessment of the Predictive Value of Selected Scales

**Figure 1:**
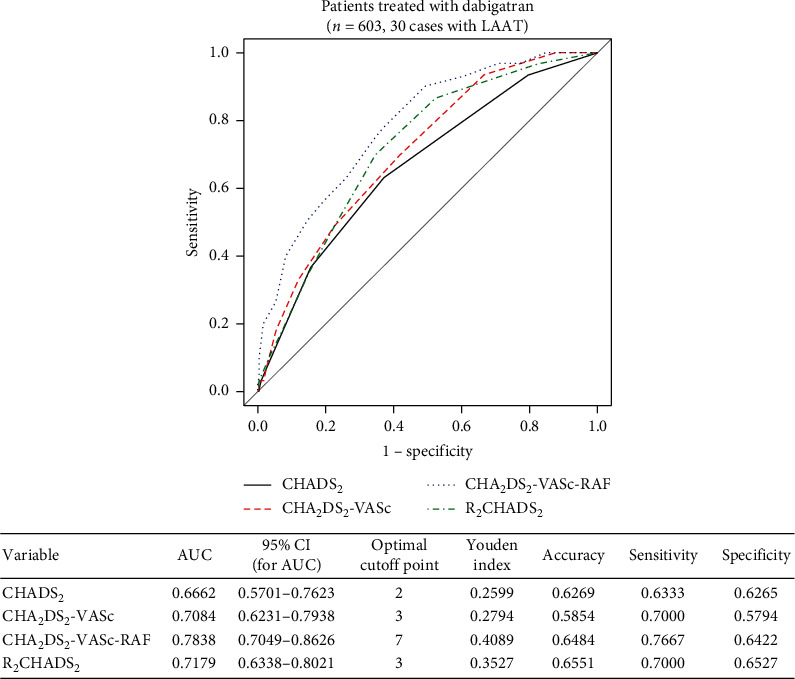
Receiver operating curves of CHADS_2_, CHA_2_DS_2_-VASc, CHA_2_DS_2_-VASc-RAF, and R_2_CHADS_2_ for predicting LAAT and associated characteristics of that prediction in the dabigatran treatment group.

**Figure 2:**
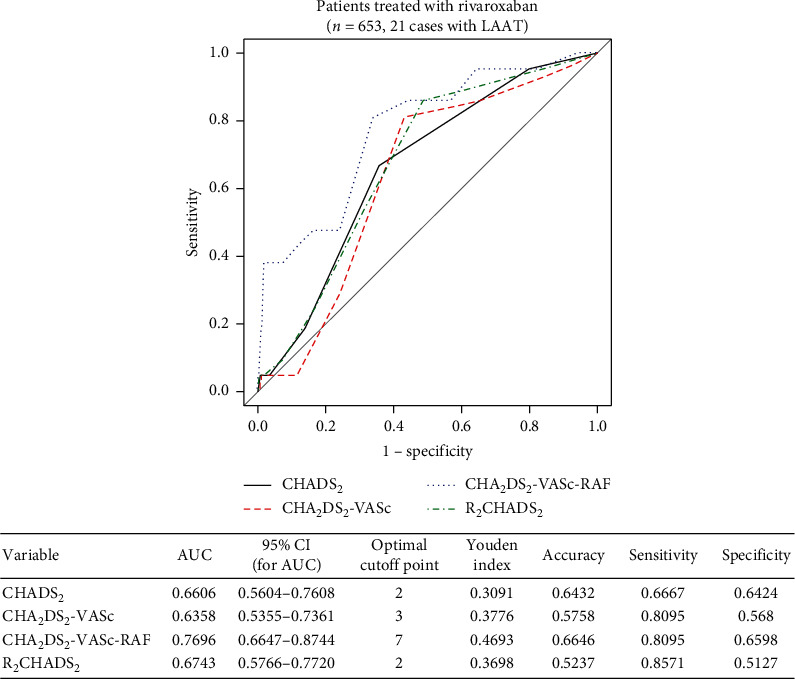
Receiver operating curves of CHADS_2_, CHA_2_DS_2_-VASc, CHA_2_DS_2_-VASc-RAF, and R_2_CHADS_2_ for predicting LAAT and associated characteristics of that prediction in the rivaroxaban treatment group.

ROC curves corresponding to the discriminant capacity of selected scales indicated that the CHA_2_DS_2_-VASc-RAF scale had better predictive ability for LAAT than any of the other presented scales in both the dabigatran and rivaroxaban treatment groups (Figures 1 and 2).

Based on the Youden index, the optimal cutoffs for the CHADS_2_, CHA_2_DS_2_-VASc, CHA_2_DS_2_-VASc-RAF, and R_2_CHADS scores to predict LAAT are 2 points, 3 points, 7 points, and 3 points, respectively (Figures 1 and 2). The results are the same for each treatment group.

In the group of patients treated with dabigatran, the CHA_2_DS_2_-VASc-RAF score showed statistically significant higher ability to differentiate between patients with LAAT and without LAAT compared to the CHA_2_DS_2_-VASc score (*p*=0.0189) and CHADS_2_ (*p*=0.0036) score, whereas the difference compared to the R_2_CHADS_2_ score (*p*=0.0875) was statistically insignificant (*p*=0.0875).

Similarly, in the group of patients treated with rivaroxaban, the CHA_2_DS_2_-VASc-RAF score showed statistically significant higher ability to differentiate between patients with LAAT and without LAAT compared to the CHA_2_DS_2_-VASc score (*p*=0.0071) and CHADS_2_ score (*p*=0.0498), whereas the difference compared to the R_2_CHADS_2_ score was statistically indifferent (*p*=0.07).

Both in the cases of patients treated with dabigatran and with rivaroxaban, the differences between remaining pairs of scores in terms of differentiating between LAAT presence and its lack were not statistically significant (*p* > 0.05).

## 4. Discussion

The risk for LAAT is similar in patients treated with dabigatran and rivaroxaban, irrespective of the dose. In both treatment groups, heart failure and non-paroxysmal AF (vs paroxysmal AF) proved to be strong predictors of LAAT. Impaired renal function was a predictor of LAAT only in patients treated with dabigatran.

Despite continuous NOAC treatment, 5% of patients treated with dabigatran and 3.2% of patients treated with rivaroxaban developed LAAT. The percentage of SEC was statistically lower in patients treated with dabigatran vs. rivaroxaban (12.6% vs. 18.8%, *p*=0.0025).

In a group of 611 patients with AF or atrial flutter before catheter ablation, Wu et al. showed that the incidence of LAAT was 3% in patients treated with dabigatran and 3.5% in patients treated with rivaroxaban [15]. Bertaglia et al. analysed a group of 414 AF patients treated with NOACs prior to electrical cardioversion or catheter ablation and found that LAAT was diagnosed in 3.1% of those treated with dabigatran and 4.7% of those treated with rivaroxaban [9]. In the X-VeRT study of patients treated with rivaroxaban, LAAT occurred in 2.7% of 564 patients who underwent TOE prior to electrical cardioversion of AF [16].

Compared to our study, the frequency of LAAT in the RE-LY trial was lower: 1.8% in the dabigatran 110 mg BID (twice daily dosing) group and 1.2% in the dabigatran 150 mg BID group [17]. In the RE-LY study, a low percentage of patients underwent TOE before electrical cardioversion. TOE was conducted for 26% of patients treated with the reduced dose of dabigatran and 24% of patients treated with the full dose [17].

In our study, a reduced dose of NOACs was prescribed to 8.6% of patients treated with dabigatran and 8.9% of patients treated with rivaroxaban. A reduced NOAC dose was not a predictor of LAAT in patients treated with dabigatran or rivaroxaban. Similarly, Gawalko et al. [18] reported that prior to cardioversion or catheter ablation, 8% of patients treated with dabigatran and 11% of patients treated with rivaroxaban were prescribed reduced doses and did not show an increased risk of LAAT. In our study, 26% of patients constituted those from the study of Gawalko et al. [18]. A meta-analysis by Reers et al. [6] showed that the approximately 5% rate of LAAT formation is considerably higher than the average stroke rate of <1%. This suggests that not all thrombi present during or after electrical cardioversion cause a cerebral thromboembolic event.

Some studies have found the CHADS_2_ and CHA_2_DS_2_-VASc scores to be the strongest predictors of LAAT prior to electrical cardioversion [19–21].

In our study, the CHADS_2_ and CHA_2_DS_2_-VASc scores were statistically higher in patients with LAAT than those without, regardless of whether they were treated with dabigatran or rivaroxaban. In the logistic regression analysis, individual components of the CHADS_2_ and CHA_2_DS_2_-VASc scales were assessed because different patients with the same score on these scales can each present with a completely different clinical picture. As most components of the CHADS_2_ and CHA_2_DS_2_-VASc scores are risk factors for atherosclerosis, the atherothrombotic mechanism may explain the positive association of CHADS_2_/CHA_2_DS_2_-VASc scores with stroke events in patients with nonvalvular atrial fibrillation (NVAF). In the era of NOACs, Frenkel et al. [22] showed that no patients with a CHA_2_DS_2_-VASc score of 0 and normal ejection fraction (EF) (≥55%) had LAAT. Kawabata et al. [23] also reported that LAAT was absent in patients with a CHA_2_DS_2_-VASc score of 0 or in paroxysmal AF patients without a prior stroke/transient ischaemic attack (TIA) history. Contrary to the aforementioned studies, our study found LAAT in patients with low risk of thromboembolic complications according to the abovementioned scales. Therefore, it is extremely important to look for factors that predispose to LAAT other than the CHADS_2_ and CHA_2_DS_2_-VASc scores.

In our study, non-paroxysmal AF was the strongest independent predictor of LAAT in patients treated with dabigatran and rivaroxaban (OR 6.2 for patients treated with dabigatran and OR 5.73 for patients treated with rivaroxaban). Data regarding the effect of AF on the incidence of LAAT are inconsistent. The authors of the guidelines of the European Society of Cardiology emphasise that the type of AF does not affect the risk of thromboembolic complications [3]. However, meta-analysis data from 99,969 patients with AF confirmed that the risk of thromboembolic complications in patients with non-paroxysmal AF is 38% higher than in patients with paroxysmal AF [24]. There may occur as various mechanisms by which non-paroxysmal AF carries a higher thromboembolic risk than paroxysmal AF, including changes in LA wall structure, longer periods of LAA/LA stunning, or other cardiovascular abnormalities promoting thromboembolism.

Heart failure is a recognized risk factor for thromboembolic complications in patients with AF. Univariate analysis within our study determined that heart failure was the strongest of all the CHA_2_DS_2_-VASc scale components as a predictor of LAAT in all patients, which was confirmed by multivariate analysis. It was also among the most powerful determinants of LAAT in a meta-analysis including 20,516 AF patients [25].

Impaired renal function is a recognized risk factor for thromboembolic complications. In the patients treated with dabigatran, an eGFR <60 ml/min/1.73 m^2^ was predictive for LAAT. This is clinically relevant because the patient characteristics and the percentage of patients with eGFR <60 ml/min/1.73 m^2^ in both NOAC treatment groups were similar. The meta-analysis including 538,479 patients showed that renal impairment is an independent risk factor for thromboembolic events (relative risk of 1.62) and postulated that it should be added to the existing risk scores [26]. In our study, the CHA_2_DS_2_-VASc-RAF scale, taking into account the presence of eGFR <56 ml/min/1.73 m^2^, was the best predictor of LAAT in patients treated with either NOAC.

Our study demonstrated that LAAT would be detectable even in patients undergoing NOAC therapy. It might suggest that TOE should be performed in all patients with AF before electrical cardioversion or catheter ablation; however, the limitations of these invasive procedures, their potential side effects, and the personnel and equipment limitations of centres performing these procedures are known. The European Heart Rhythm Association (EHRA) conducted a survey in 54 centres to examine contemporary clinical practice regarding preprocedural diagnostic work-up in AF patients; only 6 centres (12%) routinely performed TOE prior to left atrial procedures, regardless of the type or duration of AF [27]. Therefore, we are constantly looking for predictors to identify patients treated with NOAC who have a high risk of LAAT and for whom TOE is necessary. Based on the results of our study, patients treated with dabigatran who have non-paroxysmal AF, heart failure, or eGFR<60 ml/min/1.73 m^2^ and patients treated with rivaroxaban who have non-paroxysmal AF or heart failure should undergo TOE examination before electrical cardioversion or catheter ablation because they are at an increased risk of LAAT.

### 4.1. Study Limitations

While investigating predisposing factors for LAAT formation in AF patients, attempts were made to identify factors predisposing to thromboembolic complications. In AF patients, the LAA is the most common source of embolism; so, the presence of LAAT can be considered a predisposing factor for thromboembolic complications. A limitation of the study is its retrospective nature; data such as detailed echocardiographic data (e.g., LAAV and EF) were not available for all subjects; therefore, they were not included in the multivariate analysis. The number of patients treated with apixaban was too small to be included in the analysis. Another limitation was the inability to determine the AF burden; the duration of arrhythmia in the subjects was unknown, which did not allow us to distinguish between persistent and long-standing persistent AF.

## 5. Conclusions

Despite consistent anticoagulant therapy, LAAT was diagnosed in AF patients and occurred with the same frequency in those treated with dabigatran and rivaroxaban. Periprocedural TOE should be performed in all patients with non-paroxysmal AF and heart failure and in those with eGFR <60 ml/min/1.73 m^2^ if they are being treated with dabigatran. The new CHA_2_DS_2_-VASc-RAF scale had the highest predictive value for LAAT in the entire study group. Its use is worth considering in patients with AF treated with a NOAC who are to undergo TOE prior to electrical cardioversion or catheter ablation.

## Figures and Tables

**Table 1:** Scales applied to estimate the risk of thromboembolic complications in patients with AF.

Risk factors	Scales			
	CHADS_2_ (maximum score 6)	R_2_CHADS_2_ (maximum score 8)	CHA_2_DS_2_-VASc (maximum score 9)	CHA_2_DS_2_-VASc-RAF (maximum score 21)
Congestive heart failure	1	1	1	1

Hypertension	1	1	1	1

Diabetes mellitus	1	1	1	1

Vascular disease	—	—	1	1

Age 65–74 years	—	—	1	1

Stroke or transient ischaemic attack	2	2	2	2

Age ≥75 years	1	1	2	2

Female sex	—	—	1	1

Creatinine clearance <60 mL/min	—	2	—	—

eGFR <56 mL/min/1.73 m^2^	—	—	—	2

Persistent AF	—	—	—	4

Permanent AF	—	—	—	10

Risk categories				

Low	0	0	0 points in men,	0–4 points in men,
			1 point in women	1–5 points in women

Intermediate	1	1	1 point in men,	—
			2 points in women	

High	2	2	≥ 2 points in men,	≥ 5 points in men,
			≥ 3 points in women	≥ 6 points in women

AF, atrial fibrillation; eGFR, estimated glomerular filtration rate.

**Table 2:** Baseline characteristics of patient study groups.

Variable	All patients (*n* = 1256)	Patients treated with dabigatran (*n* = 603)	Patients treated with rivaroxaban (*n* = 653)	*p*
Female, *n* (%)	479 (38.1)	215 (35.7)	264 (40.4)	0.0818
Age, mean ± SD	62.5 ± 11.4	62.2 ± 11.3	62.7 ± 11.6	0.3245
Median (IQR), years	64.0 (57.0, 70.0)	63.0 (56.0, 69.0)	64.0 (57.0, 70.0)	
Clinical characteristics, *n* (%)				
Heart failure	263 (20.9)	131 (21.7)	132 (20.2)	0.5110
Hypertension	911 (72.5)	438 (72.6)	473 (72.4)	0.9362
Diabetes mellitus	231 (18.4)	119 (19.7)	112 (17.2)	0.2378
Stroke/TIA/peripheral thrombus	93 (7.4)	51 (8.5)	42 (6.4)	0.1707
Vascular disease	254 (20.2)	128 (21.2)	126 (19.3)	0.3945
AF type				
Paroxysmal	517 (41.2)	229 (38.0)	288 (44.1)	0.0365
Persistent	674 (53.7)	336 (55.7)	338 (51.8)	
Permanent	65 (5.2)	38 (6.3)	27 (4.1)	
Non-paroxysmal	739 (58.8)	374 (62.0)	365 (55.9)	0.0275
Thromboembolism risk				
CHADS_2_, mean ± SD Median (IQR)	1.4 ± 1.1	1.4 ± 1.1	1.4 ± 1.1	0.7413
	1.0 (1.0, 2.0)	1.0 (1.0, 2.0)	1.0 (1.0, 2.0)	
CHADS_2_ = 0, *n* (%)	247 (19.7)	119 (19.7)	128 (19.6)	0.7413
CHADS_2_ = 1, *n* (%)	536 (42.7)	251 (41.6)	285 (43.6)	
CHADS_2_ ≥ 2, *n* (%)	473 (37.7)	233 (38.6)	240 (36.8)	
CHA_2_DS_2_-VASc, mean ± SD Median (IQR)	2.4 ± 1.7	2.5 ± 1.7	2.4 ± 1.6	0.9358
	2.0 (1.0, 3.0)	2.0 (1.0, 3.0)	2.0 (1.0, 3.0)	
CHA_2_DS_2_-VASc = 0, *n*(%)	135 (10.7)	63 (9.6)	72 (11.9)	0.1947
CHA_2_DS_2_-VASc = 1, *n*(%)	280 (22.3)	122 (20.2)	158 (24.2)	
CHA_2_DS_2_-VASc ≥ 2, *n*(%)	841 (67)	418 (69.3)	423 (64.8)	
CHA_2_DS_2_-VASc-RAF mean ± SD	5.5 ± 3.6	5.7 ± 3.6	5.3 ± 3.6	0.0351
Median (IQR)	5.0 (3.0, 8.0)	6.0 (3.0, 8.0)	5.0 (2.0, 7.0)	
R_2_CHADS_2_, mean ± SD Median (IQR)	2.0 ± 1.5	2.0 ± 1.6	1.9 ± 1.5	0.2755
	2.0 (1.0, 3.0)	2.0 (1.0, 3.0)	1.0 (1.0, 3.0)	
Bleeding risk				
HASBLED, mean ± SD Median (IQR)	1.4 ± 0.9	1.4 ± 1.0	1.4 ± 0.9	0.9782
	1.0 (1.0, 2.0)	1.0 (1.0, 2.0)	1.0 (1.0, 2.0)	
HASBLED < 3	1104 (87.9)	520 (86.2)	584 (89.4)	0.0826
Laboratory tests				
HGB, mean ± SD Median (IQR)	*n* = 1229	*n* = 589	*n* = 640	0.9563
	14.2 ± 1.5	14.2 ± 1.6	14.2 ± 1.5	
	14.2 (13.2, 15.2)	14.2 (13.2, 15.2)	14.2 (13.3, 15.1)	
PLT, mean ± SD Median (IQR)	*n* = 1225	*n* = 586	*n* = 639	0.0150
	221.5 ± 62.0	217.6 ± 59.6	225.1 ± 64.1	
	217.0 (179.0,253.0)	210.5 (178.0, 248.8)	222.0 (180.0, 258.0)	
Creatinine, mean ± SD Median (IQR)	1.1 ± 0.3	1.1 ± 0.3	1.1 ± 0.3	0.0324
	1.0 (0.9, 1.2)	1.1 (0.9, 1.2)	1.0 (0.9, 1.2)	
eGFR, mean ± SD	72.2 ± 17.9	70.9 ± 17.0	73.5 ± 18.6	0.0031
Median (IQR)	72.0 (58.2, 90.0)	70.3 (58.0, 87.1)	74.5 (58.5, 90.0)	
eGFR < 60 ml/min/1.73 m^2^	359 (28.6)	175 (29.0)	184 (28.2)	0.7409
Echocardiographic findings				
LA, mean ± SD Median (IQR), mm	*n* = 516	*n* = 263	*n* = 253	0.0124
	44.9 ± 5.7	45.6 ± 5.8	44.3 ± 5.6	
	45.0 (41.0, 48.0)	45.0 (42.0, 49.0)	44.0 (41.0, 48.0)	
LVDD, mean ± SD Median (IQR), mm	*n* = 412	*n* = 219	*n* = 193	0.0007
	51.7 ± 6.4	52.7 ± 6.4	50.6 ± 6.2	
	51.0 (47.0, 55.2)	53.0 (48.0, 56.0)	50.0 (46.0, 54.0)	
LVEF, mean ± SD Median (IQR), %	*n* = 616	*n* = 291	*n* = 325	0.0563
	54.4 ± 9.6	53.6 ± 10.0	55.1 ± 9.2	
	58.0 (50.0, 60)	55.0 (50.0, 60)	58.0 (50.0, 60)	
LAAV, mean ± SD median (IQR), cm/sec	*n* = 769	*n* = 319	*n* = 450	0.0062
	0.5 ± 0.3	0.5 ± 0.3	0.5 ± 0.3	
	0.4 (0.3, 0.7)	0.5 (0.3, 0.7)	0.4 (0.3, 0.6)	
Study endpoint				
LAAT, *n* (%)	51 (4.1)	30 (5.0)	21 (3.2)	0.1145

AF, atrial fibrillation; eGFR, estimated glomerular filtration rate; HGB, hemoglobin; LA, left atrial; LAAT, left atrial appendage thrombus; LAAV, left atrial appendage peak emptying velocity; LVEF, left ventricular ejection fraction; LVDD, left ventricular diastolic dimension; PLT, platelets; SD, standard deviation; SEC, spontaneous echocardiographic contrast; TIA, transient ischaemic attack.

**Table 3:** Comparison of patients with and without left atrial appendage thrombus among patients treated with dabigatran and rivaroxaban.

Variable	Patients treated with dabigatran (*n* = 603)		*p*	Patients treated with rivaroxaban (*n* = 653)		*p*
	With LAAT (*n* = 30)	Without LAAT (*n* = 573)		With LAAT (*n* = 21)	Without LAAT (*n* = 632)	
Female, *n* (%)	15 (50.0)	200 (34.9)	0.0924	7 (33.3)	257 (40.7)	0.5006
Age, mean ± SD Median (IQR), years	68.9 ± 8.6	61.8 ± 11.3	0.0008	67.0 ± 8.9	62.5 ± 11.6	0.0590
	68.0 (65.0, 74.0)	63.0 (56.0, 69.0)		70.0 (61.0, 73.0)	64.0 (57.0, 70.0)	
NOAC reduced dose, *n* (%)	5 (16.7)	47 (8.2)	0.1685	56 (8.9)	56 (8.9)	0.7091
Clinical characteristics, *n* (%)						
Heart failure	16 (53.3)	115 (20.1)	<0.0001	11 (52.4)	121 (19.1)	0.0009
Hypertension	22 (73.3)	416 (72.6)	0.93	18 (85.7)	455 (72.0)	0.1663
Diabetes mellitus	12 (40.0)	107 (18.7)	0.042	6 (28.6)	106 (16.8)	0.2325
Stroke/TIA/embolism peripheral	3 (10.0)	48 (8.4)	0.7334	2 (9.5)	40 (6.3)	0.6388
Vascular disease	10 (33.3)	118 (20.6)	0.0962	5 (23.8)	121 (19.1)	0.5766
AF type, *n* (%)						
Paroxysmal	2 (6.7)	227 (39.6)	<0.0001	2 (9.5)	286 (45.3)	<0.0001
Persistent	21 (70.0)	315 (55.0)		11 (52.4)	327 (51.7)	
Permanent	7 (23.3)	7 (23.3)		19 (3.0)	8 (38.1)	
Non-paroxysmal	28 (93.3)	346 (60.4)	0.0003	19 (90.5)	346 (54.7)	0.0012
Thromboembolism risk						
CHADS_2,_ mean ± SD median (IQR)	2.1 ± 1.2	1.4 ± 1.1	0.0013	1.9 ± 1.0	1.3 ± 1.1	0.0081
	2.0 (1.0, 3.0)	1.0 (1.0, 2.0)		2.0 (1.0, 2.0)	1.0 (1.0, 2.0)	
CHADS_2_ = 0, *n* (%)	2 (6.7)	117 (20.4)	0.0127	1 (4.8)	127 (20.1)	0.0165
CHADS_2_ = 1, *n* (%)	9 (30.0)	242 (42.2)		6 (28.6)	279 (44.1)	
CHADS_2_ ≥ 2, *n* (%)	19 (63.3)	214 (37.3)		14 (66.7)	226 (35.8)	
CHA_2_DS_2_-VASc, mean ± SD Median (IQR)	3.7 ± 1.7	2.4 ± 1.7	<0.0001	3.0 ± 1.4	2.4 ± 1.6	0.0309
	3.5 (2.0, 5.0)	2.0 (1.0, 3.0)		3.0 (3.0, 4.0)	2.0 (1.0, 3.0)	
CHA_2_DS_2_-VASc = 0, *n* (%)	0 (0.0)	72 (12.6)	0.0078	1 (4.8)	62 (9.8)	0.2026
CHA_2_DS_2_-VASc = 1, *n* (%)	2 (6.7)	120 (20.9)		2 (9.5)	156 (24.7)	
CHA_2_DS_2_-VASc ≥ 2, *n* (%)	28 (93.3)	381 (66.5)		18 (85.8)	414 (65.5)	
CHA_2_DS_2_-VASc-RAF, mean ± SD Median (IQR)	9.7 ± 4.1	5.5 ± 3.5	<0.0001	9.5 ± 4.6	5.2 ± 3.5	<0.0001
	9.5 (7.0, 11.8)	5.0 (3.0, 8.0)		7.0 (7.0, 13.0)	5.0 (2.0, 7.0)	
R_2_CHADS, mean ± SD Median (IQR),	3.2 ± 1.5	1.9 ± 1.5	<0.0001	2.8 ± 1.5	1.9 ± 1.5	0.0051
	3.0 (2.0, 4.0)	2.0 (1.0, 3.0)		3.0 (2.0, 3.0)	1.0 (1.0, 3.0)	
Bleeding risk						
HASBLED, mean ± SD Median (IQR)	1.9 ± 1.0	1.4 ± 1.0	0.0107	1.9 ± 1.2	1.4 ± 0.9	0.0822
	2.0 (1.0, 2.0)	1.0 (1.0, 2.0)		2.0 (1.0, 2.0)	1.0 (1.0, 2.0)	
HASBLED < 3	24 (80.0)	496 (86.6)	0.2833	17 (81.0)	567 (89.7)	0.2645
Laboratory tests						
eGFR, mean ± SD	62.8 ± 18.9	71.3 ± 16.9	0.0076	69.5 ± 20.6	73.6 ± 18.6	0.5156
Median (IQR)	59.6 (50.6, 76.6)	70.8 (58.3, 87.4)		71.3 (52.0, 90.0)	74.6 (58.9, 90.0)	
eGFR < 60 ml/min/1.73 m^2^	16 (53.3)	159 (27.7)	0.0026	9 (42.9)	175 (27.7)	0.1285

AF, atrial fibrillation; eGFR, estimated glomerular filtration rate; LAAT, left atrial appendage thrombus; NOAC, non-vitamin K antagonist oral anticoagulants; SD, standard deviation; TIA, transient ischaemic attack.

**Table 4:** Univariate and multivariate analysis of predictive factors for LAAT in patients treated with dabigatran.

Variable	Univariate analysis			Multivariate analysis		
	OR	95% CI	*p*	OR	95% CI	*p*
Heart failure	4.55	2.16–9.6	0.0001	3.22	1.50–6.95	0.003

Hypertension	1.04	0.45–2.38	0.93			

Age (per 5 years)	1.40	1.15–1.70	0.0009			

Diabetes mellitus	2.90	1.36–6.21	0.006			

Stroke/TIA/peripheral thrombus	1.22	0.36–4.15	0.7558			

Vascular disease	1.93	0.88–4.23	0.1015			

Female	1.86	0.89–3.89	0.097			

Non-paroxysmal AF	9.18	2.17–3.89	0.0026	6.20	1.43–26.93	0.015

eGFR < 60 ml/min/1.73 m^2^	2.98	1.42–6.24	0.0039	2.65	1.24–5.66	0.012

95% CI, 95% confidence interval; AF, atrial fibrillation; eGFR, estimated glomerular filtration rate; OR, odds ratio; TIA, transient ischaemic attack.

**Table 5:** Univariate and multivariate analysis of predictive factors for LAAT in patients treated with rivaroxaban.

Variable	Univariate analysis			Multivariate analysis		
	OR	95% CI	*p*	OR	95% CI	*p*
Heart failure	4.65	1.93–11.19	0.0006	3.19	1.30–7.85	0.0116

Hypertension	2.33	0.68–8.02	0.1783			

Age (per 5 years)	1.21	0.98–1.50	0.0822			

Diabetes mellitus	1.98	0.75–5.23	0.1657			

Stroke/TIA/peripheral thrombus	1.56	0.35–6.92	0.5603			

Vascular disease	1.32	0.47–3.67	0.5953			

Female	0.73	0.29–1.83	0.5			

Non-paroxysmal AF	7.85	1.81–33.99	0.0058	5.73	1.29–25.58	0.0221

eGFR < 60 ml/min/1.73 m^2^	1.96	0.81–4.73	0.1351			

95% CI, 95% confidence interval; AF, atrial fibrillation; eGFR, estimated glomerular filtration rate; OR, odds ratio; TIA, transient ischaemic attack.

## Data Availability

The source data used to support the findings of this study are available from the corresponding author upon request.

## References

[1] Lip G. Y. H., Freedman B., De Caterina R., Potpara T. S. (2017). Stroke prevention in atrial fibrillation: past, present and future. *Thrombosis and Haemostasis*.

[2] Bukowska A., Hammwöhner M., Corradi D., Mahardhika W., Goette A. (2018). Atrial thrombogenesis in atrial fibrillation. *Herzschrittmachertherapie + Elektrophysiologie*.

[3] Kirchhof P., Benussi S., Kotecha D. (2016). ESC Guidelines for the management of atrial fibrillation developed in collaboration with EACTS. *Europace*.

[4] Klein A. L., Grimm R. A., Murray R. D. (2017). Use of transesophageal echocardiography to guide cardioversion in patients with atrial fibrillation. *The New England Journal of Medicine*.

[5] Arnold A. Z., Mick M. J., Mazurek R. P., Loop F. D., Trohman R. G. (1992). Role of prophylactic anticoagulation for direct current cardioversion in patients with atrial fibrillation or atrial flutter. *Journal of the American College of Cardiology*.

[6] Reers S., Karanatsios G., Borowski M. (2018). Frequency of atrial thrombus formation in patients with atrial fibrillation under treatment with non-vitamin K oral anticoagulants in comparison to vitamin K antagonists: a systematic review and meta-analysis. *European Journal of Medical Research*.

[7] McCready J. W., Nunn L., Lambiase P. D. (2010). Incidence of left atrial thrombus prior to atrial fibrillation ablation: is pre-procedural transoesophageal echocardiography mandatory?. *Europace*.

[8] Kosmalska K., Rzyman M., Miękus P. (2019). Usefulness of transesophageal echocardiography before cardioversion in atrial arrhythmias. *Journal of Cardiology*.

[9] Bertaglia E., Anselmino M., Zorzi A. (2017). NOACs and atrial fibrillation: incidence and predictors of left atrial thrombus in the real world. *International Journal of Cardiology*.

[10] Schulman S. (2017). Advantages and limitations of the new anticoagulants. *Journal of Internal Medicine*.

[11] Gage B. F., Waterman A. D., Shannon W., Boechler M., Rich M. W., Radford M. J. (2001). Validation of clinical classification schemes for predicting stroke. *JAMA*.

[12] Lip G. Y. H., Halperin J. L. (2010). Improving stroke risk stratification in atrial fibrillation. *The American Journal of Medicine*.

[13] Piccini J. P., Stevens S. R., Chang Y. (2013). Renal dysfunction as a predictor of stroke and systemic embolism in patients with nonvalvular atrial fibrillation. *Circulation*.

[14] Kapłon-Cieślicka A., Budnik M., Gawałko M. (2019). Atrial fibrillation type and renal dysfunction as important predictors of left atrial thrombus. *Heart*.

[15] Wu M., Gabriels J., Khan M. (2018). Left atrial thrombus and dense spontaneous echocardiographic contrast in patients on continuous direct oral anticoagulant therapy undergoing catheter ablation of atrial fibrillation: comparison of dabigatran, rivaroxaban, and apixaban. *Heart Rhythm*.

[16] Cappato R., Ezekowitz M. D., Klein A. L. (2014). Rivaroxaban vs. vitamin K antagonists for cardioversion in atrial fibrillation. *European Heart Journal*.

[17] Connolly S. J., Ezekowitz M. D., Yusuf S. (2009). Dabigatran versus warfarin in patients with atrial fibrillation. *New England Journal of Medicine*.

[18] Gawałko M., Kapłon-Cieślicka A., Budnik M. (2017). Comparison of different oral anticoagulant regimens in patients with atrial fibrillation undergoing ablation or cardioversion. *Polish Archives of Internal Medicine*.

[19] Bejinariu A. G., Härtel D. U., Brockmeier J., Oeckinghaus R., Herzer A., Tebbe U. (2016). Left atrial thrombi and spontaneous echo contrast in patients with atrial fibrillation. *Herz*.

[20] Melduni R. M., Gersh B. J., Wysokinski W. E. (2018). Real-time pathophysiologic correlates of left atrial appendage thrombus in patients who underwent transesophageal-guided electrical cardioversion for atrial fibrillation. *The American Journal of Cardiology*.

[21] Barysiene J., Zebrauskaite A., Petrikonyte D D. (2017). Findings of transoesophageal echocardiogram in appropriately anticoagulated patients with persistent atrial fibrillation prior to planned cardioversion. *BMC Cardiovascular Disorders*.

[22] Frenkel D., D’Amato S. A., Al-Kazaz M. (2016). Prevalence of left atrial thrombus detection by transesophageal echocardiography. *JACC: Clinical Electrophysiology*.

[23] Kawabata M., Goya M., Sasaki T. (2017). Left atrial appendage thrombi formation in Japanese non-valvular atrial fibrillation patients during anticoagulation therapy. *Circulation Journal*.

[24] Ganesan A. N., Chew D. P., Hartshorne T. (2016). The impact of atrial fibrillation type on the risk of thromboembolism, mortality, and bleeding: a systematic review and meta-analysis. *European Heart Journal*.

[25] Di Minno M. N., Ambrosino P., Dello Russo A A. (2016). Prevalence of left atrial thrombus in patients with non-valvular atrial fibrillation. A systematic review and meta-analysis of the literature. *Thromb Haemost*.

[26] Zeng W. T., Sun X. T., Tang K. (2015). Risk of thromboembolic events in atrial fibrillation with chronic kidney disease. *Stroke*.

[27] Farkowski M. M., Jubele K., Marín F. (2020). Diagnosis and management of left atrial appendage thrombus in patients with atrial fibrillation undergoing cardioversion or percutaneous left atrial procedures: results of the European Heart Rhythm Association survey. *EP Europace*.

